# Performance of unanchored matching-adjusted indirect comparison (MAIC) for the evidence synthesis of single-arm trials with time-to-event outcomes

**DOI:** 10.1186/s12874-020-01124-6

**Published:** 2020-09-29

**Authors:** Yawen Jiang, Weiyi Ni

**Affiliations:** 1 School of Public Health (Shenzhen), Sun Yat-sen University, Room 215, Mingde Garden #6, 132 East Outer Ring Road, Pan-yu District, Guangzhou, Guangdong China; 2 Department of Pharmaceutical and Health Economics, University of Southern California, 635 Downey Way, Verna & Peter Dauterive Hall (VPD) Suite 210, Los Angeles, CA 90089-3333 USA

**Keywords:** Matching-adjusted, Indirect comparison, Reconstruction: single-arm, Evidence synthesis

## Abstract

**Background:**

The objectives of the present study were to evaluate the performance of a time-to-event data reconstruction method, to assess the bias and efficiency of unanchored matching-adjusted indirect comparison (MAIC) methods for the analysis of time-to-event outcomes, and to propose an approach to adjust the bias of unanchored MAIC when omitted confounders across trials may exist.

**Methods:**

To evaluate the methods using a Monte Carlo approach, a thousand repetitions of simulated data sets were generated for two single-arm trials. In each repetition, researchers were assumed to have access to individual-level patient data (IPD) for one of the trials and the published Kaplan-Meier curve of another. First, we compared the raw data and the reconstructed IPD using Cox regressions to determine the performance of the data reconstruction method. Then, we evaluated alternative unanchored MAIC strategies with varying completeness of covariates for matching in terms of bias, efficiency, and confidence interval coverage. Finally, we proposed a bias factor-adjusted approach to gauge the true effects when unanchored MAIC estimates might be biased due to omitted variables.

**Results:**

Reconstructed data sufficiently represented raw data in the sense that the difference between the raw and reconstructed data was not statistically significant over the one thousand repetitions. Also, the bias of unanchored MAIC estimates ranged from minimal to substantial as the set of covariates became less complete. More, the confidence interval estimates of unanchored MAIC were suboptimal even using the complete set of covariates. Finally, the bias factor-adjusted method we proposed substantially reduced omitted variable bias.

**Conclusions:**

Unanchored MAIC should be used to analyze time-to-event outcomes with caution. The bias factor may be used to gauge the true treatment effect.

## Introduction

Comparative effectiveness evidence is essential for clinical decision and formulary policy making, heath technology assessments, and economic evaluations. When direct comparisons and network meta-analyses (NMA) are infeasible, population-adjusted indirect comparison methods may be used for evidence syntheses of comparative effectiveness [1]. Such methods include matching-adjusted indirect comparison (MAIC), simulated treatment comparisons (STC), and multi-level network meta regression (MLNMR) [1, 2], among which MAIC is relatively popular [1, 3]. The process of conducting an MAIC has been described extensively in a number of previous studies [3–5]. At its core, the MAIC method utilizes the individual-level patient data (IPD) from the trial of an intervention (usually a manufacturer’s own product) and the published aggregate data from the trial of a comparator intervention, and re-weights the patients with IPD such that their characteristics are balanced with those of the patients from the aggregate data of the comparator’s trial [3]. The weights can be obtained using propensity scores estimated with method of moments or entropy balancing, either of which is calculated using the observed characteristics that need to be balanced [3, 5]. The outcome of the patients with IPD calculated with re-weighting is then compared with that of the published aggregate data to obtain the relative effect [3].

MAIC has received increasing popularity in the evidence-based medicine community and health technology assessment agencies [1, 6, 7]. It has been mostly implemented in an anchored approach in which a common comparator, such as the placebo group, is available across trials [6]. The relative effect in the analyses of time-to-event outcomes or survival analyses in anchored MAIC, usually quantified as hazard ratio (HR), is calculated by taking the ratio of HRs from different trials or the difference of logHRs [8]. Because of the common comparator, anchored MAIC estimates are theoretically not biased by the existence of unbalanced prognostic variables that are not effect modifiers [6]. In the less frequently used unanchored MAIC approach, a common comparator is not available and the outcomes from the re-weighted IPD and the published aggregate data must be compared directly. Hence, a key difference of unanchored MAIC from anchored MAIC is that the former compares outcomes across trials whereas the latter conceptually compares treatment effect across trials. However, at least two additional complexities arise in the unanchored analyses of time-to-event outcomes that may potentially nullify the properties of anchored MAIC. First, unbalanced prognostic variables can themselves contribute to the outcome and may become confounders without adjustment. Realizing such potential drawbacks, Phillipo et al. recommended that unanchored MAIC was not always advisable [1]. Second, HRs are estimated using regression techniques such as Cox regression that requires the IPD of the published study instead of the published aggregate data, which distinguished itself from unanchored MAIC for linear-scale outcomes in which the aggregate outcomes were compared after reweighting. Such data is typically not available to researchers and is obtained through reconstruction of digitized Kaplan-Meier (K-M) curves [9, 10]. For ease of distinction, we refer to the reconstructed IPD using digitized K-M curves as RIKM hereinafter.

Previous discussion of and studies on the properties of MAIC have focused on anchored analyses of linear-scale outcomes [3–6]. The properties of unanchored MAIC in the context of time-to-event analysis have not been investigated so far yet the literature is gradually picking up the approach without appreciating the unique profiles of this estimator [11–13]. This represents a major methodological gap that needs to be filled. Specifically, single-arm trials accounted for 50% of all US Food and Drug Administration (USFDA) accelerated hematology and oncology approvals in 2015, which continued to surge to 80% in 2018 [14]. In light of this, it is expected that more comparative effectiveness studies and economic evaluations have to utilize unanchored MAIC on time-to-event outcomes at the absence of common comparators. However, due to the two aforementioned complexities, several questions that are related to the properties of unanchored MAIC on time-to-event outcomes remain to be answered. First, does RIKM represent the original survival data well enough? This is the premise that unanchored MAIC based on RIKM can be used for indirect comparison. Although this has been partially addressed when the data reconstruction method was originally proposed, the validation of data reproducibility was only conducted by comparing the summary measures of survival data underlying one single graph to the summary measures of the reconstructed version of the same graph [9]. Surprisingly, there has been an absence of attempt to validate the reconstruction method using simulation, which was likely due to the requirement of labor-intensive manual operation. Specifically, such a simulation analysis involves digitizing the curve of each repetition that mandates manually defining the coordinates, identifying the curve, and exporting the data. This process is unlike typical simulation studies that can be fully automated with programming. Hence, a simulation-based evaluation of the performance of the reconstruction approach is needed to verify its utility. Second, what are the properties of unanchored MAIC on time-to-event outcomes with respect to bias and efficiency in different scenarios? For example, is it unbiased if all prognostic variables are captured in the creation of weight as in the case of linear outcomes regardless of fundamentally different statistical processes? Also, is it unbiased if prognostic factors and effect modifiers are unbalanced across trials? A simulation study may be effective to reveal its performance in such scenarios. Third, is there a statistical approach to estimate the boundary of the true effect if unanchored MAIC estimates are indeed biased by unbalanced and unobserved covariates? We examined if the concept of bias factor that is borrowed from observational cohort studies can be used for this purpose. To answer such questions, we conducted a simulation study to investigate the properties of unanchored MAIC on time-to-event outcomes in different scenarios. The results can shed light on the above-mentioned issues and help to guide the appropriate use of the unanchored MAIC on time-to-event outcomes.

## Methods

### Simulated data

Two scenarios were simulated to investigate the properties of unanchored MAIC on time-to-event outcomes. Under each scenario, hypothetical data of breast cancer patients were simulated for two single-arm trials. For simplicity, the interventions in the trials were called treatment A and B, respectively. It was assumed that researchers had access to the IPD of treatment A but not to that of comparator B. The purpose of unanchored MAIC was, therefore, to compare the effectiveness of B versus A. The outcome in the trials was recurrence-free survival time (RFS), which was defined as the time from the start of the intervention to the earlier of all-cause death and disease recurrence. In both scenarios, unbalanced covariates across trials were simulated by design. In the present study, an effect modifier was defined as a variable that interacted with the intervention in the data generation of time to event and a prognostic factor was a variable that itself loaded on time to event. In the first scenario, effect modifiers of treatments were not included. The same set of prognostic factors were simulated for each arm (or trial), which were age, an indicator variable for menopausal status (postmenopausal vs. not), and indicator variables for tumor grades (1, 2, and 3). The B arm data also contained an indicator of treatment B. The estimated effect of B in relation to A using unanchored MAIC was captured by the HR associated with this indicator. The prognostic factors of the A arm were set so such that the patients in the A arm were in less severe conditions than the B arm. In other words, the A arm had lower average values of the prognostic factors that were negatively associated with RFS. Age was a continuous variable and was simulated using normal distributions truncated at the lower and upper bounds, while the other prognostic factors were indicators simulated using Bernoulli distributions. The specifications of the distributions used for the prognostic factors of the two arms are listed in Table 1. The sample sizes of the A and B arms were arbitrarily set at 1000 and 800, respectively.

**Table 1:** Parameters used in the simulation of the A and B arms

	A arm	B arm
Sample size	1000	800
Mean age	53	56
Standard deviation of age	11	12
Range of age	[21, 80]	[23, 85]
Menopausal status (% post-menopausal)	40	50
tumor grade = 1 (%)	20	10
tumor grade = 2 (%)	65	70
tumor grade = 3 (%)	15	20
Distribution	Weibull	Weibull
Scale parameter	0.00000004	0.00000004
Shape parameter	2.2	2.2
Coefficients of variables (logHR)		
Age (yearly increment)	0.02	0.02
Menopausal status	0.5	0.5
Indicator variable for tumor grade = 2	0.3	0.3
Indicator variable for tumor grade = 3	0.6	0.6
B indicator	NA	−0.5 (in the scenario without effect modifier) / -0.4 (in the scenario with effect modifier)
B indicator × menopausal status interaction	NA	−0.2 (only applicable to the scenario with effect modifier)
Maximum follow-up time (days)	2500	2500

Abbreviation: *HR* Hazard ratio; *NA* Not applicable

The RFS were simulated using Weibull distributions. The shape and scale parameters of the Weibull distribution and the coefficients of the prognostic factors in the linear component of the Weibull distribution are displayed in Table 1. Random censoring was included based on a uniform distribution that was truncated at 2500 days. In the second scenario, not only the prognostic factors were unbalanced but also menopausal status was both a prognostic factor and an effect modifier of B. There was no change to the A arm. Hence, the A arm was directly taken from the simulation in scenario 1. Whereas the coefficients of the other prognostic factors remained the same in the B arm across the scenarios, that of the B indicator was changed from − 0.5 in the first scenario to − 0.4 in the second scenario. In addition, an interaction term of the B indicator and menopausal status was included in the linear component to incorporate the modification effect. The coefficient of the interaction term was set at − 0.2 such that the expected treatment effect was the same across the two scenarios. The simulated data in the B arms were used to generate aggregate characteristics and K-M curves, which correspond to the published data of typical single-arm clinical trials. One-thousand sets of triplet time-to-event data (one for the A arm, one for the B arm without any effect modifiers, and one for the B arm with an effect modifier) were simulated. Subsequent analyses of the statistical performance of unanchored MAIC using different analytic strategies were conducted between the A arm and the B arms within each set. The results from the 1000 repetitions formed the distributions of the estimates using alternative MAIC strategies which are described later. As mentioned previously, each repetition in the present study involved digitizing the hypothetically published K-M curve of the B arm and required heavy manual operation. Hence, the number of repetitions was restricted to 1000.

### Validation of digitization-based reconstruction method

The validation of the reconstruction method was based on comparison of the 1000 repetitions of RIKM and the simulated data for the generation of the curves. RIKM of both B arms (with and without the effect modifier) were compared to the corresponding simulated raw data using Cox regressions with an indicator of being reconstructed data and a variable representing the time-varying effect of the “being reconstructed” indicator. The hypotheses were that the HRs of the indicator and the time-varying effect would both equal one if the reconstructed data sufficiently mirrored the raw data. To quantify the assessment, the mean HRs were estimated as

$$ {\overline{\mathrm{HR}}}_{\mathrm{k}}^{rc}=\frac{1}{\mathrm{N}}\sum \limits_{j=1}^N\left(\hat{{\mathrm{HR}}_{\mathrm{j},\mathrm{k}}^{rc}}\right) $$

and

$$ {\overline{\mathrm{HR}}}_{\mathrm{k}}^{tv}=\frac{1}{\mathrm{N}}\sum \limits_{j=1}^N\left(\hat{{\mathrm{HR}}_{\mathrm{j},\mathrm{k}}^{tv}}\right) $$

where ${\overline{\mathrm{HR}}}_{\mathrm{k}}^{rc}$ and ${\overline{\mathrm{HR}}}_{\mathrm{k}}^{tv}$ are respectively the mean HRs of the reconstruction indicator and the time-varying effect, N is the number of repetitions in each scenario, and $\hat{{\mathrm{HR}}_{\mathrm{j},\mathrm{k}}^{rc}}$ and $\hat{{\mathrm{HR}}_{\mathrm{j},\mathrm{k}}^{tv}}$ are respectively the estimated HRs of the reconstruction indicator and the time-varying effect from the jth repetition of the kth (1st or 2nd) scenario. Also, the percentages of the 95% confidence intervals (CIs) that covered one were calculated for both estimates in both scenarios. It was expected that the percentages were at least 95%.

### Strategies of unanchored MAIC on time-to-event outcomes

The general data analytic steps of unanchored MAIC are 1) balancing the IPD with the aggregate data to obtain weights; 2) digitization for RIKM; and 3) pooling the IPD and the RIKM to conduct weighted survival analysis. Two methods have been proposed to balance the prognostic factors of the IPD data with those of aggregate data, namely propensity score matching using a method-of-moments logistic regression and entropy balancing [1, 15]. Phillipo et al. noticed that the two methods are equivalent in reducing bias yet the latter generates smaller standard errors [1]. Also, entropy balancing generates equal weighted sample sizes of the two groups [16]. In the present study, entropy balancing was used to balance the prognostic factors and to obtain weights. Both the mean and the variance of age were used for balancing. This reflects real-world practice because the variance of characteristics such as that of age is usually reported in the publication of clinical trials. The other covariates including the indicator of menopausal status and the indicators of tumor grades were balanced on the percentages. These variables were dichotomous variables of which the balance of the second moment follows that of the first moment [17]. Table 2 lists a statistical summary of the prognostic factors of the A arm before and after balancing as well as the target aggregate data of the B arm using one of the repetitions as an example. Three analytic strategies were evaluated for unanchored MAIC in the first scenario. The first strategy was an unweighted analysis ignoring the unbalanced prognostic factors across trials. In the second strategy, all prognostic variables were included when conducting entropy balancing to create weight. In the third strategy, the indictors for menopausal status and tumor grades were omitted in the creation of weight.

**Table 2:** Example of entropy balancing

	A arm		B arm
	Pre-balance	Post-balance	Aggregate data (target of balance)
Mean age (years)	52.55	56.14	56.14
Variance of age	125.3	143.0	142.9
Postmenopausal (%)	40.00	52.12	52.13
Tumor grade 2^a^ (%)	65.20	72.86	72.88
Tumor grade 3^a^ (%)	14.40	18.37	18.38

^a^ Tumor grade 1 was a left-out reference group

In the second scenario, four analytic strategies were evaluated. Similar to the first scenario, the first and second strategies were an unweighted analysis and a weighted analysis using all prognostic factors, respectively. The third strategy was a weighted analysis omitting the effect modifier (menopausal status) in the creation of weight, while the fourth strategy further dropped tumor grade indicators from the balanced variable list.In all analyses, Cox regressions were used to estimate the HRs, and the comparison of strategies was based on logHRs.

### Performance of unanchored MAIC

To quantify the performance of unanchored MAIC in the analysis of time-to-event outcomes, the bias, the Monte-Carlo variance (MCV), the mean squared error (MSE), and the percentages of CI coverage of the estimates were evaluated for each strategy. Among these, MCV is the squared of the empirical standard errors [18], which is a measure of the efficiency of the estimator. The bias was calculated as

$$ \frac{1}{\mathrm{N}}\sum \limits_{j=1}^N\left(\log \hat{{\mathrm{HR}}_{\mathrm{j}}}-\left(-0.5\right)\right), $$

the MCV was calculated as

$$ \frac{1}{\mathrm{N}-1}\sum \limits_{j=1}^N{\left(\log \hat{{\mathrm{HR}}_{\mathrm{j}}}-\log \overline{\mathrm{HR}}\right)}^2, $$

and the MSE was calculated as

$$ \frac{1}{\mathrm{N}}\sum \limits_{j=1}^N{\left(\log \hat{{\mathrm{HR}}_{\mathrm{j}}}-\left(-0.5\right)\right)}^2 $$

where $\hat{{\mathrm{HR}}_{\mathrm{j}}}$ is the estimated HR from the jth repetition in each scenario and $\overline{\mathrm{HR}}$ is the mean of $\hat{{\mathrm{HR}}_{\mathrm{j}}}$ over N repetitions.

In addition to these quantities, the effective sample size (ESS) was also calculated [19]. Although not an indicator of the performance of MAIC estimates, ESS was informative in that its value should be close to the true sample size of the B arm when the characteristics of the two arms were balanced without having to rely on extreme weights [3]. A flowchart of the overall process of simulation, analysis, and comparison is illustrated in Fig. S1. Engauge Digitizer 10.11 [20] was used to digitize the K-M curves (screenshots of digitizing and exporting displayed in Figs. S2, S3, S4). Reconstruction of RIKM was implemented using Stata ipdfc routine and all statistical analyses were conducted using Stata 14 (StataCorp LLC, College Station, Texas, the United States of America) [10].

### Using the bias factor to estimate the boundary of the true effect

For an exposure E, an unmeasured dichotomous confounder U, and an outcome D, VanderWeele et al. has shown that

$$ \frac{H{R}_{obs}}{H{R}_{true}}\le bias\ factor, $$

where *HR*_*obs*_ is the observed effect and *HR*_*true*_ is the true effect. The bias factor is calculated as

$$ \left(H{R}_{UD}\times R{R}_{EU}\right)/\left(H{R}_{UD}+R{R}_{EU}-1\right) $$

where *HR*_*UD*_ is the maximal possible effect of U on D and *RR*_*EU*_ is the risk ratio of U = 1 of the exposed group to the non-exposed group [21, 22]. As such, the inequality $H{R}_{true}\ge \frac{H{R}_{obs}}{bias\ factor}$ suggests that *HR*_*true*_ should not be smaller than $\frac{L{L}_{\hat{HR}}}{bias\ factor}$ in 95% of the repetitions in which $L{L}_{\hat{HR}}$ is the lower limit of the 95% CI. If so, the strongest plausible effect can be estimated using *HR*_*UD*_ and *RR*_*EU*_. The former can be estimated using the IPD of the trial that the researchers can access, the latter can be based on assumptions or external sources. We calculated $\frac{\hat{HR}}{bias\ factor}\  and\frac{L{L}_{\hat{HR}}}{bias\ factor}$ for the two scenarios by setting menopausal status as U, following which we summarized the mean bias of $\log \frac{\hat{HR}}{bias\ factor}$ and the percentages of the repetitions in which $\frac{L{L}_{\hat{HR}}}{bias\ factor}$ was smaller than the true value. By the set-up of the data simulation, the bias factor was 1.10 and 1.05 in the two scenarios, respectively (the calculation was illustrated in online supplementary materials part II).

## Results

The results of comparing the raw data and RIKM of the B arms as a validation of the reconstruction method are listed in Table 3. A graphical example of a raw survival curve and the counterpart using the digitization and reconstruction method is presented in Fig. S5. The mean HRs of the recovered indicator in the first and the second scenarios were correspondingly 0.959 and 0.960, whereas the mean HRs of time-varying effect in both scenarios were 1.00. Also, the percentages of repetitions in which the 95% CI covered one were 100% in both scenarios.

**Table 3:** Agreement between the raw data and the reconstructed data of the B arms

	Scenario 1	Scenario 2
HR of recovered vs. raw indicator	0.959	0.960
Coverage of the 95% CI of the HR of indicator (% of repetitions)	100	100
HR of time-varying effect	1.000	1.000
Coverage of the 95% CI of time-varying effect (% of repetitions)	100	100

Abbreviations: *HR* Hazard ratio; *CI* 95% Confidence interval

The results of the performance evaluation of unanchored MAIC for survival outcomes in scenario 1 are presented in Table 4. In the first scenario, which did not involve any effect modifiers, the bias of the logHRs using the unweighted Cox regressions was 0.164. By contrast, the bias of the weighted Cox regressions that used all prognostic factors in entropy balancing was substantially smaller at 0.027. Although less than the unweighted analyses, the bias of the weighted analyses when the indicators of menopausal status and tumor grades were dropped from entropy balancing was 0.114. More, the MCV of the estimates was the same across all analytic strategies, which was 0.002. Even more, the MSEs of the three analytic strategies were 0.029, 0.003 and 0.015, respectively. Finally, the percentages of repetitions in which the 95% CI covered the true value were 11.2, 93.8 and 39.1% for the unweighted, fully weighted, and partially weighted strategies. None of the coverage reached the expected 95% although the fully weighted approach reached a close approximation.

**Table 4:** Estimates of log hazard ratio in scenario 1 (without effect modifiers)

Statistical properties	Unweighted estimate	Weighted estimate	
		All variables included in weight	Omitted menopausal status and tumor grade from weight
Bias	0.164	0.027	0.114
MCV	0.002	0.002	0.002
MSE	0.029	0.003	0.015
CI coverage (%)	11.2	93.8	39.1
mean ESS	NA	791	888

Abbreviation: *MCV* Monte-Carlo variance; *MSE* Mean squared error; *CI* 95% Confidence interval; *ESS* Effective sample size

The performance evaluation results related to scenario 2 are listed in Table 5. In the second scenario, the unweighted analysis had a bias of 0.173. The fully weighted analyses had a bias of 0.035. In addition, the bias of the weighted analyses omitting the effect modifier was 0.079. More, the weighted analyses omitting both the indicator of menopausal status and the indicators of tumor grades had a bias of 0.122. The MCV of the unweighted estimator and the weighted approach omitting both the indicator of menopausal status and the indicators of tumor grades was 0.002 whereas that of the other two analytic strategies was 0.003. The MSEs of these four analytic strategies were 0.032, 0.004, 0.009 and 0.017, respectively. The percentages of repetitions in which the 95% CI covered the true value were 7.7, 89.9, 68.7 and 34.1% for the four strategies correspondingly. Similar to scenario 1, the fully weighted approach in scenario 2 was the closest to the threshold of 95% but had an even greater shortage in coverage compared with scenario 1.

**Table 5:** Estimates of log hazard ratio in scenario 2 (with an effect modifier)

Statistical properties	Unweighted estimate	Weighted estimate		
		All variables included in weight	Omitted the effect modifier (menopausal status) from weight	Omitted the effect modifier (menopausal status) and tumor grade from weight
Bias	0.173	0.035	0.079	0.122
MCV	0.002	0.003	0.003	0.002
MSE	0.032	0.004	0.009	0.017
CI coverage (%)	7.7	89.9	68.7	34.1
mean ESS	NA	791	827	888

Abbreviation: *MCV* Monte-Carlo variance; *MSE* Mean squared error; *CI* 95% Confidence interval; *ESS* Effective sample size

By the study design, the ESS of the fully weighted approach was the same in the two scenarios. Specifically, the ESS was 791 when all covariates were balanced, which was close to the true sample size of the B arm. As expected, the ESS was greater when the list of covariates for balancing was shorter.

The performance of adjustment methods using bias factors are presented in Table 6. The mean bias of the bias factor-adjusted HRs in the log scale was − 0.025 and 0.030 in the two scenarios, respectively. The magnitude of bias of the adjusted HRs were comparable to that of the fully weighted approaches in both scenarios. The corresponding percentages of repetitions of which the true value was not less than the adjusted lower limit (LL) were 93.3 and 91.8%, respectively. These percentages were close to but did not reach the expectation of 95%.

**Table 6:** Bias factor-adjusted HR and lower limits of the 95% CI of HR using menopausal status as an omitted variable

	Scenario 1	Scenario 2
Mean bias of adjusted HR in the log scale	−0.025	0.030
Adjusted LL of the CI of HR ≤ true effect (% of repetitions)	93.3	91.8

Abbreviations: *HR*, hazard ratio; *CI* 95% Confidence interval; *LL* Lower limit

## Discussion

In the present analysis, we examined the performance of alternative unanchored MAIC approaches to analyze time-to-event outcomes under the scenarios with and without an effect modifier. The results contribute to the information basis for the appropriate use of unanchored MAIC.

With a simulation, the present study confirmed that RIKM using the method proposed by Guyot et al. may sufficiently represent the raw time-to-event data [9]. This finding has two practical implications. First, secondary analyses using reconstructed IPD is a viable solution when raw data cannot be accessed. Second, and in a reversed perspective, studies on properties of methods related to reconstructed IPD may rely on simulated raw data instead of reconstructed data.

Our findings also revealed several important properties of unanchored MAIC. First and foremost, unanchored MAIC does have the potential to generate unbiased estimates when used to analyze time-to-event outcomes if all factors that impact either the outcome or the treatment effect are captured. That is, not only the effect modifiers but also the non-effect-modifying prognostic factors have to be balanced. Consistent with intuition, dropping some of the prognostic factors in balancing causes greater bias than balancing with full information but less bias than the unweighted approach. Second, and unlike the anchored counterpart, prognostic factors are important in unanchored MAIC analysis of time-to-event outcomes even though not being effect modifiers at the same time. In our simulation analyses, bias arose when not balancing on the prognostic factors even when there were no effect modifiers. Also, omitting the effect modifier which was also a prognostic factor led to nontrivial bias in the scenario of having an effect modifier. On top of bias, the confidence interval estimates of this approach was far from acceptable. As such, the results of the second scenario indicate that balancing both prognostic factors and effect modifiers is crucial in unanchored MAIC on time-to-event outcomes. Third, there may be a trade-off between bias and precision, yet the fully weighted approach constantly outperformed other approaches when MSE was used to evaluate the methods whereas the unweighted approach consistently ranked the worst. Therefore, the benefit of reducing bias with the fully weighted approach outweigh the precision loss in the setting of the present simulation analysis. Fourth, the uncertainty of unanchored MAIC may be biased or underestimated even when the bias of the relative effect is not a prominent problem because the coverage of the CIs never reached 95% across all strategies. This property of MAIC has not been spotted in literature previously and should be discussed in future applications of unanchored MAIC on time-to-event outcomes. The reasons of this property could be multifaceted. The loss of information in the data reconstruction step, although minuscule, may have contributed to this. More, entropy balancing followed by a Cox regression may not have fully accounted for bias. The compound of these sources of uncertainty may result in the imperfect confidence interval estimates.

In addition, we proposed an approach to estimate the boundary of the true effect when unanchored MAIC on time-to-event outcomes is likely biased due to omitted covariates. Simulation results showed that the proposed method was imperfect but were not necessarily unusable. This approach involves calculating the bias factor, which requires knowledge or assumptions of the extent of omitted variable unbalance (*RR*_*EU*_). In practice, the extent of omitted variable unbalance may be unknown in most situations. Possible solutions including using external data to estimate a plausible value of *RR*_*EU*_ or calculating the adjusted HRs and the boundaries by toggling *RR*_*EU*_ over a possible range. For example, if the percentages of post-menopausal individuals among breast cancer patients range from 35 to 65% across different trials and observational studies, then the range can be used to obtain the extremes of *RR*_*EU*_ estimates, and, for that matter, the boundaries of bias factor-adjusted treatment effect estimates. Of note, when the treatment effect is suspected to be overestimated rather than underestimated, the upper limit should be multiplied by the bias factor to estimate the boundary.

Several limitations should be noted when interpreting the results. First, we only used Weibull distribution to simulate the data sets. The data generation process in the real world may not necessarily approximate a Weibull distribution. Especially, survival curves in oncology are sometimes characterized by a high death rate due to nonresponse at the beginning or a plateau at the tail due to cure [23], which may not be sufficiently represented by single-index survival functions. As such, the generalizability of our findings is possibly limited due to the specific situations. Second, only 1000 repetitions were conducted for each scenario of data generation due to the labor-demanding process of manually completing part of the K-M curve digitization. Although the number of repetitions matches that of a previous simulation study in the realm of MAIC [5], the possibility of insufficient repetitions to reveal the properties could not be fully ruled out. Third, the same specification of shape and scale parameters of the Weibull distribution was used in the simulation of both A and B arms, which may be reasonable if the populations are adequately homogenous across trials. However, the scenarios of different underlying survival distributions across trials were not probed. Such complexity almost infinitely complicates the examination and discussion of any evidence synthesis methods. Fourth, the scenarios we explored were not exhaustive. For example, the coefficient specifications of covariates, the differences in the prognostic factors across trials, and the treatment effect were not extensively varied to examine the performance of unanchored MAIC under other scenarios. Such practice was largely hampered by the hefty manual work required to digitize the graphs. A byproduct of the limited number of scenarios for characteristic differences was that the impact of extreme weights due to larger differences in the IPD and the aggregate data could not be investigated, which was also reflected by the ESS of the fully weighted approach. Finally, censoring was simulated using a uniform distribution that was unrelated to the treatment, the effect modifier, and the prognostic factors. How non-random censoring impacts the performance of unanchored MAIC and MAIC in general in the analysis of time-to-event outcomes should be investigated in future.

## Conclusions

Reconstructed IPD from digitized K-M curves may sufficiently represent the raw time-to-event data. Also, unanchored MAIC may be used in the analysis of time-to-event outcomes across single-arm trials. However, it should be used with caution of unmeasured prognostic factors and effect modifiers as well as suboptimal CIs. More, the bias factor-adjusted estimate can be used as an approximation of the boundary of the true effect at the presence of omitted variables.

## Supplementary information

**Additional file 1.**

## Data Availability

The study did not collect primary data. Program code files used for data simulation and analyses in the submitted work can be accessed at 10.17632/6dvrxd7xpn.2.

## Abbreviations

CI: Confidence interval
HR: Hazard ratio
IPD: Individual-level patient data
K-M: Kaplan-meier
LL: Lower limit
MAIC: Matching-adjusted indirect comparison
MCV: Monte-carlo variance
MSE: Mean squared error
NMA: Network meta-analyses
RR_EU_: The extent of omitted variable unbalance
RFS: Recurrence-free survival time
RIKM: Reconstructed IPD using digitized K-M curves
USFDA: US food and drug administration
