# Supplementary material for: Performance of unanchored matching-adjusted indirect comparison (MAIC) for the evidence synthesis of single-arm trials with time-to-event outcomes
Source: BMC Med Res Methodol. 2020 Sep 29;20:241. doi: 10.1186/s12874-020-01124-6 (PMC7526361; doi:10.1186/s12874-020-01124-6)
Supplement: Supplementary file 1 — Additional file 1. [file 12874_2020_1124_MOESM1_ESM.pdf]

Part I. Supplementary figures.

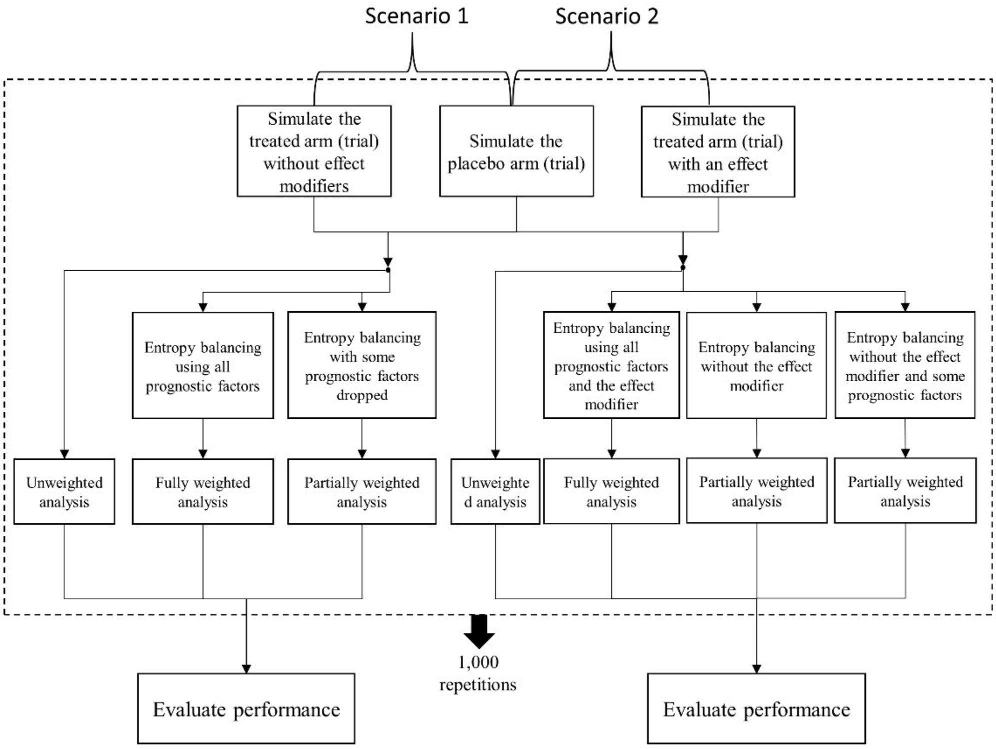

Figure S1. The flowchart of the overall data simulation, weighting, reconstruction, and analysis process.

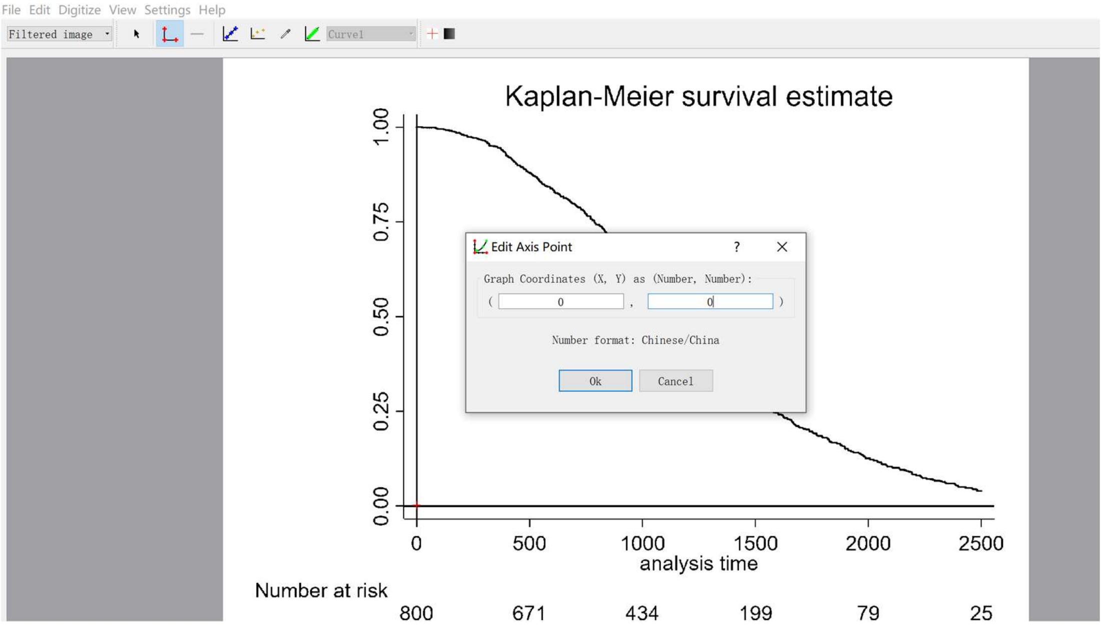

Fig S2. Defining coordinates in Engauge Digitizer. In the current screenshot, the origin was being defined. However, the coordinates can be defined using any three given points.

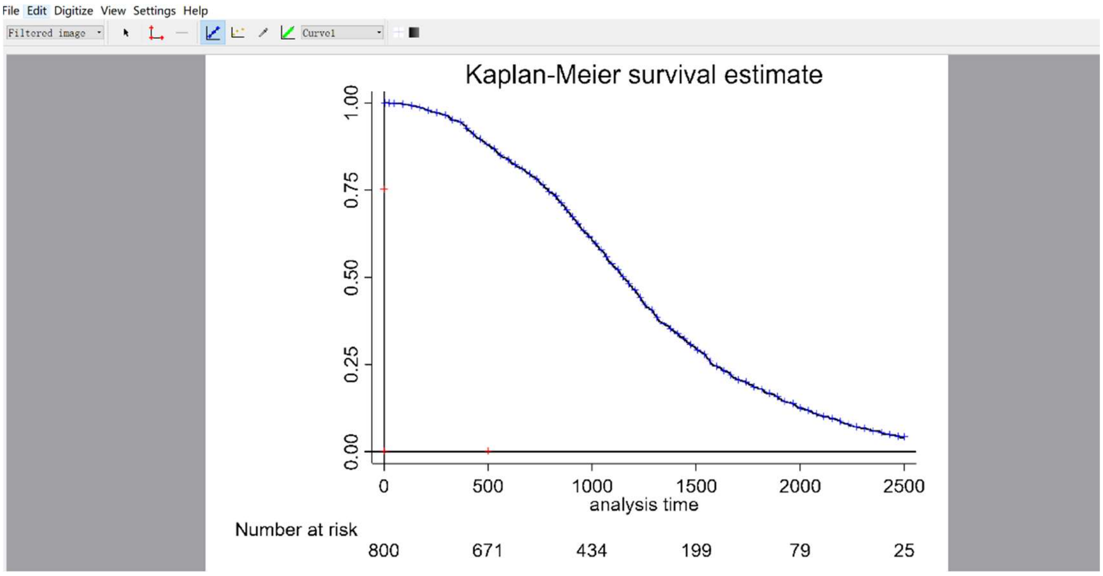

Fig S3. Selecting and digitizing the curve. In the current screenshot, a series of points on the survival curve were selected to represent the curve and digitized based on their positions in reference to the coordinates.

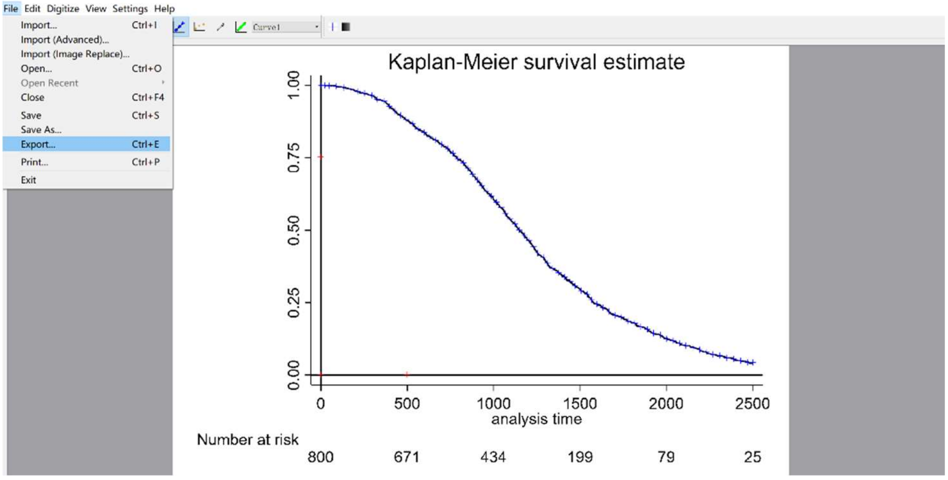

Fig S4. Exporting the digitized points as a data set. The data can be saved as a csv file, which can be imported by Excel.

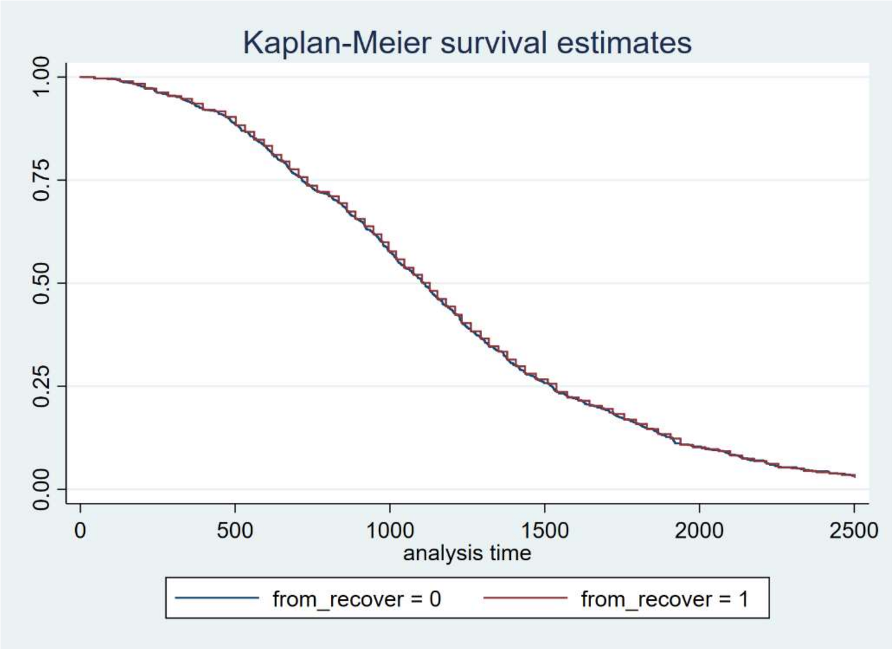

Fig S5. An example of a raw survival curve and its digitized and reconstructed counterpart. The blue line was the curve using the raw data and the red line was the curve using the reconstructed data.

Part II. Calculation of bias factors under the two scenarios

In the first scenario, in which there was no interaction effects, the coefficient of menopausal status was set as 0.5. Hence, $HR_{UD} = e^{0.5} = 1.65$. Also, the proportions of post-menopausal women were set to be 40% and 50% in the A and B arms, respectively. Therefore, $RR_{EU} = \frac{0.5}{0.4} = 1.25$. Taken together, the bias factor=$(HR_{UD} \times RR_{EU})/(HR_{UD} + RR_{EU} - 1)$=1.10. In the second scenario, the coefficient of the interaction term was -0.2. Hence, $HR_{UD} = e^{0.5-0.2} = 1.35$. As such, the bias factor=$(HR_{UD} \times RR_{EU})/(HR_{UD} + RR_{EU} - 1)$=1.05.
